# Mechanisms underlying anxiety in Rett Syndrome: Translational insights from preclinical findings

**DOI:** 10.1016/j.nsa.2022.100109

**Published:** 2022-09-18

**Authors:** Bethan Impey, Tracey A. Newman, David S Baldwin

**Affiliations:** Clinical and Experimental Sciences, Faculty of Medicine, University of Southampton, Southampton, UK

**Keywords:** Rett syndrome, *Neurobiology*, Anxiety, Epigenetics, Translational research

## Abstract

• The role of serotonin in mechanisms of anxiety in Rett syndrome appears complex.
• *Mecp2* mutation alters HPA axis function but this is understudied in Rett syndrome.
• Rett syndrome may inform understanding of anxiety in those with early life stress.

## Nomenclature:

*MECP2*: is the gene in humans
*Mecp2*: is the murine gene
MeCP2: is the protein in mouse and humans

## 1 Introduction

Rett syndrome is the second most common cause of severe learning disability in females (occurring in approximately 1:10,000 females). Babies born with Rett syndrome initially develop apparently normally, however, at the age of 6–18 months, development falters and subsequently regresses (Box 1). Rett syndrome has multiple comorbid conditions affecting overlapping physiological systems. Despite this heterogeneity, anxiety is a common feature amongst the comorbidities of Rett syndrome (Gold et al., 2017). The mechanisms underlying anxiety are the focus of this review as further study may lead to new insights into the pathophysiology of anxiety.

Box 1 **Stages of Rett syndrome (defined by Hanefeld, 1985) (Gold et al., 2017; Zhou et al., 2017)**

**Table:** 

Stage (name)	Age	Clinical features
Stage I (‘stagnation’)	6–18 months	Development slows and gain of new skills stops.
Stage II (‘regression’)	1–4 years	Rapid loss of skills that have been gained, including communication, social skills and motor skills. Development of stereotypic hand-movements. Other common features that develop are seizures, disordered breathing (with hyperventilation and apneas), gastrointestinal complications and anxiety.
Stage III (‘pseudostationary’)	2–10 years	There is a stabilisation and some skills that were lost in the regression stage may be regained.
Stage IV (‘motor deterioration’)	Can last for decades	There is a decrease in mobility, with muscle weakness or spasticity. Cognition and language skills do not alter.

Alt-text: Box 1

The challenge of identifying anxiety in individuals with severe intellectual disability has been recognised. Two validated questionnaires have been developed for identifying presumed anxiety-related behaviours in Rett Syndrome: the Rett Syndrome Behaviour Questionnaire (RSBQ) and the Anxiety, Depression and Mood Scale (ADAMS) (Esbensen et al., 2003; Mount et al., 2002). These questionnaires rely on carer observation. The RBSQ was validated by comparing results in individuals with Rett syndrome and those with other severe intellectual disability; control for physical impairment was also included (Mount et al., 2002). Anxiety symptoms in Rett syndrome appear similar to generalised anxiety and social anxiety as they include worsening of hyperventilation, inconsolable crying, trembling in the absence of fearful situations and withdrawal from social contact (Buchanan et al., 2019).

While the diagnosis of Rett syndrome is made using clinical features, it is usually caused by mutations on chromosome Xq28 in the gene encoding methyl-CpG-binding protein 2 (*MECP2*). MeCP2 is found in most tissues, but is most abundant in the central nervous system (CNS) (Shahbazian et al., 2002). A key role of MeCP2 is to bind to methylated DNA and it can both activate and repress gene expression, therefore acting as an epigenetic regulator. The effects of MeCP2 are cell-type and region specific and the level of MeCP2 within a cell is critical to the function of the cell. MeCP2 expression increases in the early months of life and the timing of expression in particular brain regions correlates with the maturation of the CNS (a time when sensory-driven neural activity shapes the CNS circuitry) (Zimmermann et al., 2015). In Rett syndrome there is mosaicism (only the maternal or paternal X chromosome will be expressed in any one cell in the body) of *MECP2* gene expression and this leads to variability in phenotype (Gold et al., 2017). Further variations in expression of the condition are likely influenced by the numerous mutations in *MECP2*; to date 518 likely pathogenic mutations have been identified.

A review of autonomic dysregulation in individuals with Rett syndrome has highlighted the complexity of treatment (Singh and Santosh, 2018). Treatments for anxiety disorders in neurotypical individuals are often suboptimal. This is further complicated in neurodivergent individuals in whom the mechanisms and treatments for anxiety are poorly understood. Symptoms suggestive of anxiety are found more commonly in Rett syndrome compared to individuals with other severe intellectual disability (Mount et al., 2002) suggesting the mechanisms of anxiety may be entwined with the disorder. Greater understanding of anxiety mechanisms in Rett syndrome could facilitate development of more specific treatments and improve our mechanistic understanding of anxiety disorders in general.

A number of preclinical models of Rett syndrome have been developed in mice; many of these show similar features to patients with Rett syndrome, including anxiety-like behaviour, irregular breathing, motor impairment and seizures (Katz et al., 2012). This review focuses on the preclinical evidence base. It highlights the heterogeneity in the mutations being studied, the study designs and the need for standardisation of behavioural assays. Despite these limitations, these models provide a system for the study of the cellular and molecular mechanisms of MeCP2 dysfunction and associated alterations in anxiety-like behaviour. The discussion draws together findings from pre-clinical studies with clinical observations and suggests future directions for Rett syndrome translational research, and postulates that there may be broader implications for other disorders of MeCP2 dysfunction where anxiety is a feature.

## 2 Methods

**Fig. 1:**
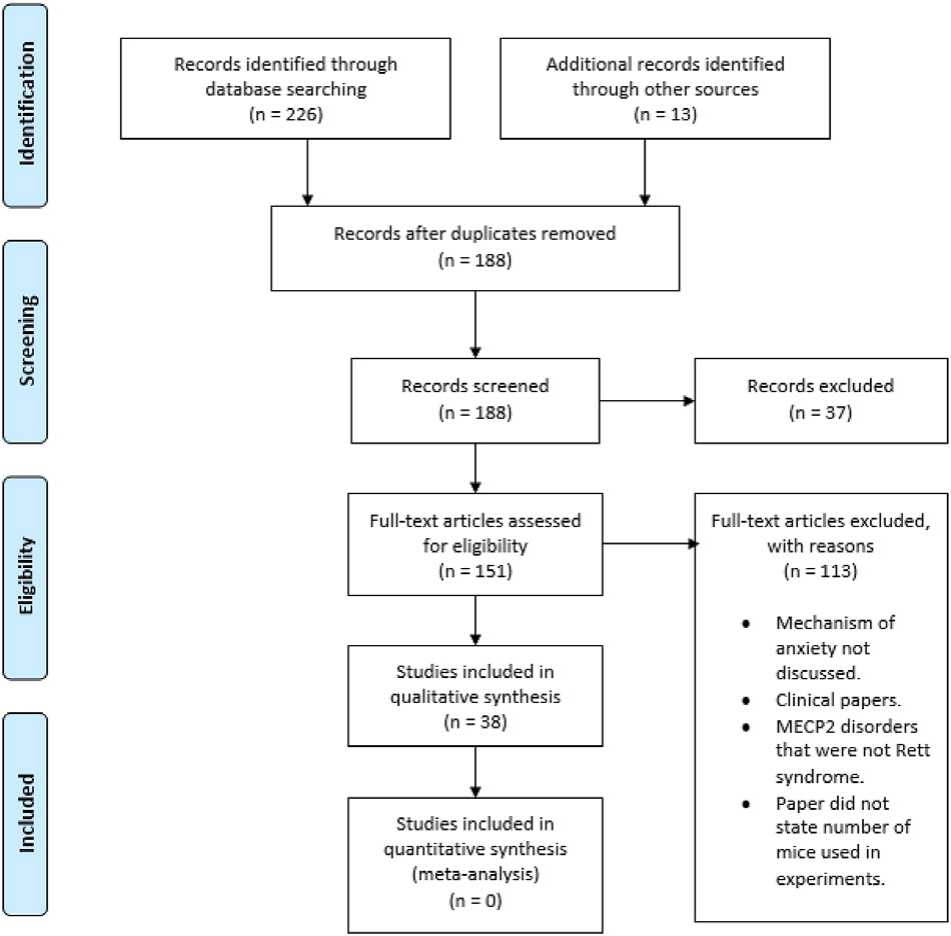
PRISMA flow chart. Records were checked for duplicates and the remaining papers were filtered. Exclusion criteria were: papers referring to disorders of MECP2 that were not Rett syndrome; papers that did not explore mechanisms of anxiety in Rett syndrome; papers that investigated scales for assessing anxiety in people with Rett syndrome. Papers analysing levels of monoamines (and their metabolites) in Rett syndrome but without attempting to correlate with anxiety-like behaviour were not included as this has already been reviewed (Roux and Villard, 2010). Clinical papers were also excluded from further analysis (due to a systematic review of clinical studies assessing autonomic dysregulation in Rett syndrome having previously been done (Singh and Santosh, 2018) there would have been overlap in the results and so clinical studies of anxiety mechanisms are referred to in the discussion. Studies were excluded where the gender or number of mice used was not clearly reported. One of the identified papers was a review of a relevant paper; the review was excluded but the original article was included. Further relevant papers were identified within the references of identified papers from the original search.

A systematic review was carried out using the Preferred Reporting Items for Systematic Review and Meta-Analysis (PRISMA) guidelines (Moher et al., 2009). Records were searched through PubMed, MEDLINE and Embase. The following search terms were used [Rett syndrome] AND [anxiety]. Searching was completed on February 3, 2022. Fig. 1 illustrates the selection process. All included studies made statements that animal experiments complied with ethical guidelines, except for McGill et al., 2006, Orefice et al., 2016 and Wither et al., 2013 (McGill et al., 2006; Orefice et al., 2016; Wither et al., 2013), where statements were not explicitly made. All included studies were published in peer-reviewed journals.

## 3 Results

### 3.1 Overview

**Fig. 2:**
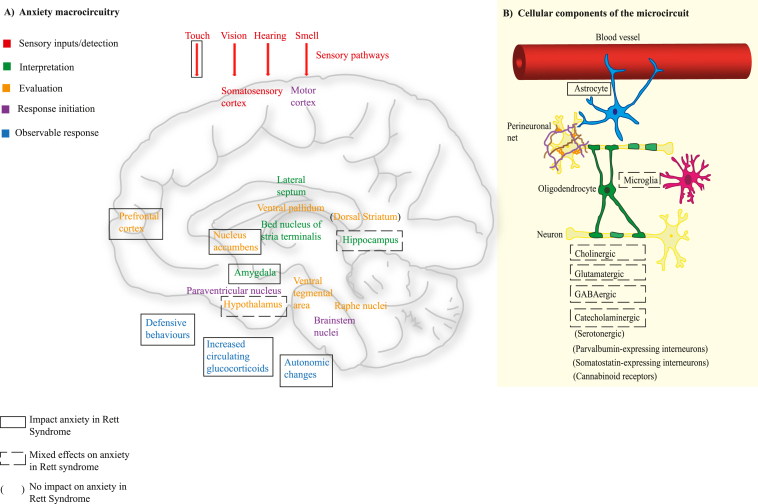
Effects of MeCP2 within anxiety macro- and micro-circuitry regions. Panel A: known anxiety macro-circuitry. Colours (see key) indicate the stages of anxiety processing within and between the anatomical regions of the circuit. The anatomical regions have projections that integrate signals associated with the interpretation and evaluation of stimuli to enable assessment of potential threats and produce a response. Panel B: cellular components that form into microcircuits through local connections and associations. The key at the bottom left identifies symbols (used within panel A and B) that summarise the findings within this paper – anatomical regions and cell types that do/do not impact anxiety-related behaviours in pre-clinical models of Rett syndrome.

Anxiety is postulated to develop when there are disruptions in the interconnected macro- and micro-circuits that process stimuli detected from the external world. As such, the first stage in the anxiety macrocircuit involves the sensory systems. Sensory signals are interpreted as potentially threatening through the interconnected activity (via neuronal projections between regions) of the amygdala, bed nucleus of the stria terminalis, ventral hippocampus and prefrontal cortex. Evaluation, where the interpreted signals are regulated, then occurs. This involves the medial prefrontal cortex, ventral tegmental area, nucleus accumbens, ventral pallidum, and hypothalamus. Following coordinated processing of a threat stimulus, a response is initiated (Calhoon and Tye, 2015). Microcircuits function within each region of the anxiety macrocircuit and involve short-range connections between neuronal subtypes and associated interactions with glial cells, the blood vessel endothelium and surrounding specialised extracellular matrix (perineuronal net). The account of the preclinical studies has been structured to highlight the effect of MeCP2 loss in anxiety macrocircuit regions and within cellular subtypes on anxiety-like behaviour and/or physiological response (illustrated within Fig. 2) and subsequently summarises potential underlying molecular mechanisms. At present, there is limited literature examining macro- and micro-circuitry connections, which is briefly described in section 3.6. However, an effect of MeCP2 loss in CNS regions and cell subtypes on anxiety behaviour may suggest an impact on the anxiety circuitry, albeit with significant limitation on interpretation.

The tests of anxiety-like behaviours are described within Box 2. Generation of the mouse models relies on mutation of the *Mecp2* alleles. In keeping with the variability of mutations identified in people with Rett syndrome, the mouse models involve varying degrees of severity of mutation – null alleles, large mutations and point mutations. For clarity, male Rett syndrome mice are referred to as ‘*Mecp2* null’ and female Rett syndrome mice are referred to as ‘*Mecp2*^+/−^‘. Most earlier Rett syndrome studies used male mice, although this is changing with the recognition of the importance of studying both sexes and the obvious importance of using female mice given that in humans, females are affected. The reason that male mice had been predominantly used is because *Mecp2* mutation causes a less severe phenotype in mice than humans and the male mice show a more pronounced phenotype than females. In addition, females have a more variable phenotype than males, due to mosaicism (Ribeiro and MacDonald, 2020). Table 1 documents studies that correlate molecular changes with anxiety behaviour; included studies are arranged alphabetically on the basis of the first author(s). This table details experimental conditions and enables changes in anxiety behaviour to be identified within and between studies, as well as highlighting whether male and/or female mice were used. Outcomes of the studies within the table are summarised in the text. The sections documenting MeCP2 loss in macrocircuit regions and cell subtypes have been structured to summarise where this was associated with a change/no change/mixed effects on anxiety behaviour. The description ‘mixed effects’ was determined by there being inconsistent changes between the different behavioural tests within a study. The impacts of MeCP2 loss within anxiety macrocircuit regions and cell subtypes are summarised within Fig. 2. Details of the mutation used in each study have been documented within Table 2.

Box 2 **Description of unconditioned tests of anxiety**

These four tests exploit mice's innate avoidance of heights, light and open spaces, which conflicts with their spontaneous exploration of novel surroundings.

*Open field* – uses a chamber that is novel to the mouse. It has lines to demarcate the floor space. This enables activity to be quantified. A mouse is placed in the centre of the chamber and allowed to explore freely. Each line crossing is counted as a unit of activity. The amount of time spent in the centre versus periphery is scored. Greater activity and greater time spent in the centre both indicate lesser anxiety. Defecation, rearing and grooming behaviour can also be used as measures of anxiety (the more frequent these behaviours, the greater the level of anxiety). Total distance travelled/total activity was not included as an anxiety measure in this review since Rett syndrome affects motor activity, providing a confounding reason for altered total activity.

*Light-dark assay* – employs an environment that is novel to the mouse. It contains a larger light and smaller dark compartment. A mouse is placed in the light compartment and allowed to explore. The number of transitions and time spent in each compartment are recorded. The greater the number of transitions and the greater the amount of time spent in the light, the less anxious the mouse. Risk assessments – when mice adopt an extended posture before crossing from dark to light compartments – may also be used as a measure of anxiety (increased risk assessment by the mouse indicate higher levels of anxiety).

*Elevated Plus maze* – this consists of four arms extending from a central platform to make a plus shape. Two arms are walled and two are open (bright and exposed area); the central platform is also open. The plus maze is elevated above the ground. The mouse is released onto the central platform and the amount of time spent in the open and closed arms is assessed; more time in the open arm indicates less anxiety. The number of risk assessments made before moving from closed to open arms can be quantified; more risk assessments indicate greater anxiety.

*Elevated zero maze* – a variation of the elevated plus maze without the central starting platform – this is thought to be an advantage as mice often stay here for some time and interpretation of this behaviour is difficult. The elevated zero maze is circular and consists of alternating open and closed arcs (Bailey and Crawley, 2009).

Other tests were employed within the studies, for example the startle response, but these have been excluded from the description in the results as the four tests above are considered the most effective tests of anxiety, as opposed to fear or a measure of social behaviour (which is defined as a separate feature of Rett syndrome). These four tests are also the most frequently used anxiety measures within the studies and aided comparability between studies. A novelty suppressed feeding test was used in one paper (Kondo et al., 2016), but excluded as it is considered a less effective test of anxiety.

N.B. There is debate regarding ‘anthropomorphisation’ of pre-clinical animal models of anxiety behaviour. We refer to ‘anxiety’-related behaviours in mice as ‘anxiety’ throughout the review but recognise the marked simplification of this approach.

Alt-text: Box 2

**Table 1:** ↑ increased ‘anxiety’ compared to control, ↓ decreased ‘anxiety’ compared to control, ↔ no significant difference compared to control, - no result). ‘/’ is used where more than one measure was used and indicated differing outcome: for example, in Adachi et al., (2009), in the open field test there was no significant difference in time in the centre, but there was a significant difference in ratio of time in centre over time in the periphery. Flox mice show 50% reduction in MeCP2 expression. Some studies used this as a control, so a potential change in anxiety behaviour may be under-represented by this (Samaco, 2008).

STUDY [REFERENCE]	*MECP2* MUTATION (AND EXPERIMENTAL CONDITIONS, WHERE RELEVANT)	MOUSE GENDER	DIFFERENCE IN ANXIETY WITH *MECP2* MUTATION±EXPERIMENTAL CONDITION COMPARED TO CONTROL (WITHOUT ADDITIONAL EXPERIMENTAL CONDITION, UNLESS OTHERWISE STATED)			
			Elevated plus maze	Light-dark assay	Open field test	Elevated zero maze
Abellán-Álvaro et al., 2021	*Mecp2*^+/−^ mice	Female	↓	–	↔	–
	*Mecp2*^+/−^ mice that were separated from their mother.	Female	↓	–	↔	–
Adachi et al., 2009 ∗ control for genetic carrier system used instead of wildtype	Specific deletion of *Mecp2* from the basolateral amygdala in mice	Male	↑	–	↑/↔	–
Bittolo et al., 2016	*Mecp2* null mice treated with (control solution),	Male	↓/↔	–	–	–
	*Mecp2* null mice treated with mirtazapine.	Male	↓/↔	–	–	–
	*Mecp2* null mice treated with desipramine.	Male	↓/↔	–	–	–
Castro et al., 2014	*Mecp2* null mice.	Male	↓	–	–	–
	*Mecp2* null mice with intraperitoneal rhIGF-1 treatment.	Male	↔	–	–	–
Chao et al., 2010	Specific deletion of *Mecp2* in GABAergic neurons.	Male	↔/↑	↔	↑	–
Chin et al., 2019	Mice with specific deletion of *Mecp2* in excitatory neurons of the mouse forebrain.	Male	–	–	↑	–
	Mice with specific deletion of *Mecp2* in excitatory neurons of the forebrain, with choline supplementation.	Male	–	–	↔	–
De Filippis et al., 2014	*Mecp2* null mice.	Male	–	↑/↓/↔	–	–
	*Mecp2* null mice with intraperitoneal injection of a 5-HT7R selective agonist.	Male	–	↔	–	–
Derecki et al., 2012	MECP2^+/−^ mice	Female	–	–	↑	–
	*Mecp2*^+/−^ mice that were irradiated to kill resident microglia and then given bone marrow transplant from wildtype mice.	Female	–	–	↔	–
Flores Gutiérrez et al., 2020	*Mecp2*^+/−^ mice treated with control solution.	Female	↓	↔/↑	↔	–
	*Mecp2*^+/−^ mice with trimmed whiskers treated with control solution.	Female	↔	–	–	–
	*Mecp2*^+/−^ mice (untrimmed whiskers) treated with mirtazapine	Female	↔	–	–	–
Fyffe et al., 2008	Selective deletion of *Mecp2* in Sim-1 expressing neurons, with associated reduction in MeCP2 expression in the paraventricular, supraoptic, and posterior hypothalamic nuclei, and in the nucleus of the lateral olfactory tract of the amygdala.	Male	–	↔	↑	–
Gemelli et al., 2006	Selective deletion of *Mecp2* in the forebrain (prefrontal cortex, striatum, nucleus accumbens, hippocampus and amygdala)	Male	↑	–	↑	–
Goffin et al., 2014 ∗loxp control used instead of wildtype	Specific deletion of *Mecp2* in forebrain glutamatergic neurons and glial cells.	Male	–	–	↔	↔
Hao et al., 2015	*Mecp2*^+/−^ mice exposed to deep brain stimulation (DBS).	Female	–	↓	↓	–
	*Mecp2*^+/−^ mice exposed to sham DBS.	Female	–	↓	↓	–
	*Mecp2*^+/−^ mice exposed to no DBS/sham DBS.	Female	–	↔	↔	–
He et al., 2014	Deletion of *Mecp2* in parvalbumin-expressing interneurons	Male	↔	↔	↔	–
Ito-Ishida et al., 2015	Deletion of *Mecp2* in parvalbumin-expressing interneurons.	Male	↔	↔	–	–
	Deletion of *Mecp2* in somatostatin-expressing interneurons	Male	↔	↔	–	–
Kerr et al., 2010	*Mecp2* null mice, 129/SvJ background, standard housed.	Male	↓/↔	–	–	–
	*Mecp2* null mice, 129/SvJ background, with environmental enrichment.	Male	↔	–	–	–
	*Mecp2* null mice, 129/SvJ and C57BL/6J mixed background, standard housed.	Male	↓/↔	–	–	–
	*Mecp2* null mice, 129/SvJ and C57BL/6J mixed background, with environmental enrichment.	Male	↔	–	–	–
Kondo et al., 2016	*Mecp2*^+/−^ mice, standard housed.	Female	↓	–	–	–
	MECP2^+/−^ mice with environmental enrichment.	Female	↔	–	–	–
Lang et al., 2012	*Mecp2* null mice.	Male	–	↓	↓	–
	*Mecp2* null mice with *Mecp2* rescue in catecholaminergic neurons.	Male	–	↓ (but significantly ↑ compared to null)	↓ (but significantly ↑ compared to null)	–
Lioy et al., 2011 ∗ compared to control for genetic carrier system instead of wildtype	*Mecp2* null mice.	Male	–	–	↑	↓
	*Mecp2* null mice with selective reintroduction of *Mecp2* in astrocytes.	Male	–	–	↑ (but significantly ↓ compared to null)	↓ (but significantly ↑ compared to null)
Mantis et al., 2009	*Mecp2* null mice with standard diet.	Male	–	↑	↔	–
	*Mecp2* null mice with restricted ketogenic diet.	Male	–	↑/↔	↔/↓/↑	–
	*Mecp2* null mice with caloric restriction.	Male	–	↔	↔/↓	–
Mellios et al., 2014	*Mecp2*^+/−^ mice.	Female	–	–	↑	–
	*Mecp2*^+/−^ mice with clenbuterol treatment.	Female	–	–	↑ (but ↓ compared to MECP2^+/−^ mice without clenbuterol)	–
Meng et al., 2016	*Mecp2* null mice.	Male	↓	↑	–	–
	Mice with specific deletion of *Mecp2* in glutamatergic neurons.	Male	↓	↑	–	–
	*Mecp2* null mice that had specific reintroduction of *Mecp2* in glutamatergic neurons.	Male	↔	↔	–	–
	*Mecp2*^+/−^ mice, age 10 weeks	Female	↓	–	–	–
	*Mecp2*^+/−^ mice with specific reintroduction of *Mecp2* in glutamatergic neurons, age 10 weeks.	Female	↔	–	–	–
	*Mecp2*^+/−^ mice, age 30 weeks	Female	↔	–	–	–
	*Mecp2*^+/−^ mice with specific reintroduction of *Mecp2* in glutamatergic neurons, age 30 weeks.	Female	↔	–	–	–
Mossner et al., 2020 ∗flox mice used as control instead of wildtype	Deletion of *Mecp2* in VIP-expressing interneurons	Male	↔	–	↔	–
	Deletion of *Mecp2* in parvalbumin-expressing interneurons.	Male	↔	–	↔	–
	Deletion of *Mecp2* in somatostatin-expressing interneurons	Male	↔	–	↔	–
	Deletion of *Mecp2* in VIP-, parvalbumin- and somatostatin-expressing interneurons	Male	↔	–	↔	–
Orefice et al., 2016 ∗controls – wildtype and mice lacking the genetic carrier system – paper was not fully clear about which controls were used in the individual experiments.	Deletion of *Mecp2* in dorsal root ganglion (DRG) and trigeminal somatosensory neurons	Male	↑	–	↑	–
		Female	–	–	↑	–
	Deletion of *Mecp2* in DRG during adult life (as opposed to early life)	Male	↔	–	↔	–
		Female	–	–	↔	–
	*Mecp2* null mice	Male	↑	–	↑	–
	*Mecp2* null mice with specific reintroduction of *Mecp2* in peripheral sensory neurons	Male	↔	–	↔	–
Ricceri et al., 2011	*Mecp2* null mice	Male	–	–	↑	–
	*Mecp2* null mice with choline supplementation (behavioural tests at postnatal day 60).	Male	–	–	↔	–
Samaco et al., 2009	Specific deletion of *Mecp2* in Th-expressing DA/NE neurons.	Male	–	↔	–	–
	Specific deletion of *Mecp2* in PC12 *ets* factor 1 (PET1)-expressing serotonergic neurons	Male	–	↔	–	–
	*Mecp2*^+/−^ mice.	Female	–	↓	↓	–
	*Mecp2*^+/−^ mice with *Mecp2* rescue in catecholaminergic neurons.	Female	–	↔	↔	–
Su et al., 2015	Specific deletion of *Mecp2* in the striatum.	Male	–	–	↔	↔ Compared to flox, not wildtype
	*Mecp2* null mice.	Male	–	–	↑	–
	*Mecp2* null mice with specific re-expression of *Mecp2* in the striatum.	Male	–	–	↑	–
	*Mecp2*^+/−^ mice.	Female	–	–	↔	–
	*Mecp2*^+/−^ mice with specific re-expression of *Mecp2* in the striatum.	Female	–	–	↔	–
Ure et al., 2016	*Mecp2* null mice.	Male	↓	↑	–	–
	*Mecp2* null mice with specific reintroduction of *Mecp2* into GABAergic neurons.	Male	↔	↔	–	–
Vigli et al., 2021	*Mecp2* null mice with intraperitoneal injection of saline.	Male	–	↑	–	–
	*Mecp2* null mice with intraperitoneal injection of cannabidiolic acid (CBDA)	Male	–	↑	–	–
Wither et al., 2013	*Mecp2*^+/−^ mice (since *Mecp2*^+/−^ mice is an X-linked gene, X inactivation means that there can be differing levels of MECP2 in particular brain regions). For this study there was no control. The degree of anxiety behaviour was correlated with MeCP2 expression in various brain regions - results are expressed as anxiety level with lower MeCP2 expression compared to higher MeCP2 expression (akin to wildtype) in each brain region.					
	Hippocampus	Female	–	↓	↓	–
	Cortex	Female	–	↔	↔	–
	Cerebellum	Female	–	↔	↔	–
	Spinal cord	Female	–	↔	↔	–
Zhang et al., 2014	*Mecp2* null mice	Male	–	↑	–	–
	*Mecp2* null mice exposed to deep-brain magnetic stimulation for 5 months.	Male	–	↔	–	–
Zhang et al., 2016	Specific deletion of *Mecp2* in neurons positive for choline acetyltransferase (ChAT, required for synthesis of acetylcholine).	Male	↓/↔	↓ ∗ comparison made with control for the genetic carrier system, not wildtype	↔ ∗ ↓ compared to control for the genetic carrier system	↓ ∗ comparison made with control for the genetic carrier system, not wildtype
	Specific deletion of *Mecp2* in neurons positive for ChAT, but with re-expression of *Mecp2* in the cholinergic neurons of the basal forebrain.	Male	↔ ∗ comparison made with control for the genetic carrier system, not wildtype	–	–	–
	Specific deletion of *Mecp2* in neurons positive for ChAT, but with re-expression of *Mecp2* in the cholinergic neurons of the caudate/putamen	Male	↓ ∗ comparison made with control for the genetic carrier system, not wildtype	–	–	–
	Selective activation of α7 nicotinic acetylcholine receptors in the hippocampus in the CA1 region of the hippocampus in mice with specific deletion of *Mecp2* in neurons positive for ChAT.	Male	↓ compared to control for Cre genetic carrier system, but↑ compared to mice with specific deletion of MECP2 in neurons positive for ChAT (without selective activation of α7 nicotinic acetylcholine receptors)	–	–	↔
Zhou et al., 2017	*Mecp2* null mice.	Male	↓	–	↑	–
	*Mecp2* null mice with selective reintroduction of *Mecp2* into cholinergic neurons.	Male	↓	–	↔	–

**Table 2:** Analysis of study validity.

Study	Is there evidence of a power calculation?	Can the number of mice used in experiments be clearly identified?	Is age of animals stated?	Was randomisation performed?	Evidence blinding was used?	What was the source of the animals? (details of genetic models found within appendix)	Model of MECP2 mutant mice	Mouse strain used	How were data from behavioural experiments captured?
Abellán-Álvaro et al., 2021	No	Yes	Yes	No	No	Wildtype and *Mecp2*^+/−^ mice obtained from The Jackson laboratory and crossed in house.	*Mecp2*^tm1.1Bird/J^	*Mecp2*^+/−^ mice were 129P2(C) and C57BL6 mixed background. Wildtype mice were C57BL/6J background.	Video tracking
Adachi et al., 2009	No	Yes	Yes	No	No	Not clear.	*Mecp2*^tm1.1Jae^	All mice were 129/BALB/c background that was backcrossed with C57BL/6 for 10 generations.	Video tracking
Bittolo et al., 2016	No	Yes	Yes	No	No	*Mecp2* null mice were obtained from The Jackson Laboratory and backcrossed on mice from Charles River. The origin of wildtype mice was not clearly stated.	*Mecp2*^tm1.1Bird^	*Mecp2* null mice were C57BL/6 and 129P2(C) mixed background and backcrossed on C57BL/6. Wildtype were C57BL/6J background.	Video tracking
Castro et al., 2014	No	Yes	Yes	No	No	Genetic cross of mouse lines from The Jackson Laboratory.	*Mecp2*^tm1.1Bird^	All mice were C57BL/6 and 129P2 mixed background, that was backcrossed with C57BL/6J.	Video tracking
Chao et al., 2010	No	Yes	Yes	No	No	Mice heterozygous for *Mecp2* mutation were obtained from Dr Adrian Bird. The source of other animals is not clearly stated.	*Mecp2*^tm1Bird^	The following strains appear to have been used (methods difficult to interpret due to multiple cross references): Viaat-Cre were FVB background, Viaat- *Mecp2*^-/y^ mice and wildtype controls were mixed FVB, C57BL/6 and S6SvEvTac strains, and flox ‘controls’ were S6SvEvTac background.	Hand-held computer
Chin et al., 2018	No	Yes	Yes	No	No	Genetic cross of mouse lines from The Jackson Laboratory. The origin of wildtype mice was not clearly stated.	*Mecp2*^tm1Bird^	*Mecp2*^flox+/y^; CaMKIIα-Cre mice were C57BL/6 and 129P2 mixed background and backcrossed to C57BL/6. Background not clearly stated for wildtype mice.	Automated system
De Filippis et al., 2013	No	Yes	Yes	No	No	*Mecp2* null mice were from The Jackson Laboratory. The origin of wildtype mice was not clearly stated.	*Mecp2*^tm1Hzo^	*Mecp2* null mice were C57BL/6J and 129S mixed background and backcrossed to C57BL/6J for at least 12 generations. Background not clearly stated for wildtype mice.	Video tracking
De Filippis et al., 2014	No	Yes for behavioural experiments; not clear for immunohistochemistry or Western blot.	Yes	No	No	*Mecp2* null mice were obtained from The Jackson Laboratory. The origin of wildtype mice was not clearly stated.	*Mecp2*^tm1**Hzo**^	*Mecp2* null mice were C57BL/6J and 129S mixed background and backcrossed to C57BL/6J for at least 12 generations. Background not clearly stated for wildtype mice.	Video tracking
Derecki et al., 2012	No	Yes	Yes	No	No	*Mecp2*^+/−^ mice were obtained from The Jackson Laboratory. The origin of wildtype mice was not clearly stated.	The models of *Mecp2*^+/−^ used in irradiation experiments was not clearly stated.	Not clearly stated for *Mecp2*^+/−^ or wildtype mice.	Video tracking
Flores Gutiérrez et al., 2020	Yes	Yes	Yes	Yes	Yes	Wildtype and *Mecp2*^+/−^ mice obtained from The Jackson laboratory and crossed in house.	*Mecp2*^tm1.1Bird/J^	*Mecp2*^+/−^ mice were 129P2(C) and C57BL6 mixed background. Wildtype mice were C57BL/6J background.	Video tracking
Fyffe et al., 2008	No	Yes	Yes	No	No	Gifts from Adrian Bird, Brad Lowell and Joel Elmquist.	Presumed to be *Mecp2*^tm1Bird^	Mixed 129SvEv and FVB background for all mice.	Computer-operated digiscan optical animal activity system (open field test). Hand held computer (light-dark assay).
Gambino et al., 2010	No	Yes (number of cells stated for electrophysiology experiments)	Age range stated	No	No	*Mecp2* null mice were obtained from The Jackson Laboratory. The origin of wildtype mice was not clearly stated.	*Mecp2*^tm1Hzo^	*Mecp2* null mice were C57BL/6J and 129S mixed background and were backcrossed to C57BL/6J. Background not clearly stated for wildtype mice.	Not relevant
Gemelli et al., 2006	No	Yes	Yes	No	Yes	*Mecp2* mutation created in house	Presumed to be *Mecp2*^tm1.1Jae^	All mice were mixed 129, BALBC mixed background and backcrossed to C57BL/6.	Video tracking
Goffin et al., 2014	No	Yes	Yes	Samples were pseudo-randomised	Yes	Genetic cross of mouse lines from The Jackson Laboratory	Presumed to be *Mecp2*^tm1.1Jae^	All mice were backcrossed on C57BL/6, but the original backgrounds were not clearly stated.	Video tracking (elevated zero maze) and beam breaks (open field test)
Hao et al., 2015	No	Yes	Yes	No	Yes	Not stated	Not stated	*Mecp2*^+/−^ mice were mixed FVB and 129 background. Background not clearly stated for wildtype mice.	Video tracking
He et al., 2014	No	yes	Yes	No	Yes	Mouse lines were obtained from The Jackson Laboratory. The origin of wildtype mice was not clearly stated.	*Mecp2*^tm1bird^	PV-MECP2^-/y^, *Mecp2*^flox/y^ and PV-Cre mice were mixed 129P2 and C57BL/6J background and backcrossed to C57BL/6J. The background of wildtype mice was not clearly stated.	Video tracking (open field test and elevated plus maze). Method of data capture was not stated for the light-dark assay
Ito-Ishida et al., 2015	No	Yes	Yes	No	No	PV-Cre and SOM-Cre mice were obtained from The Jackson Laboratory. The origin of other mice was not clearly stated.	*Mecp2*^tm1Bird^	PV- *Mecp2*^-/y^ mice and flox ‘controls’ were mixed 129S6SvEvTac and C57BL/6 background. PV-Cre mice were C57BL/6 background. Som-Cre were mixed 129S4Sv and C57BL/6 background. SOM- *Mecp2*^-/y^ mice were mixed 129S4Sv and C57BL/6 and 129S6SvEV background. Background not clearly stated for wildtype mice.	Video tracking
Kerr et al., 2010	No	Yes for qRT-PRC; no for behavioural experiments	Yes	No	No	*Mecp2* null mice were obtained from Adrian Bird laboratory, University of Edinburgh and The Jackson Laboratory. The origin of wildtype mice was not clearly stated.	*Mecp2*^tm1.1Bird^	*Mecp2* null mice were either 129S1/SvImJ background or mixed 129/SvJ and C57BL/6J background. Background not clearly stated for wildtype mice.	Human observer
Kondo 2016	No	Yes	Yes	No	No	Not clearly stated	*Mecp2*^tm1Tam^	All mice were 129sv and C57BL/6 mixed background.	Video tracking
Lang et al., 2012	No	Yes	Yes	No	No	TH-cre mice were a gift from Dr Joseph Savitt. *Mecp2* null mice were from The Jackson Laboratory and genetic crosses were performed in house. The origin of wildtype mice was not clearly stated.	*Mecp2*^tm2Bird^	*Mecp2*^tm2Bird^ is originally mixed 129P2 and C57BL/6J background and these mice were backcrossed on C57BL/6J. The original background of TH-cre is unknown; this line was backcrossed on C57BL/6. Background not clearly stated for wildtype mice.	Video tracking
Lioy et al., 2011	No	Yes	Yes	No	No	*Mecp2*^tm2Bird^ were from the Jackson laboratory. The origin of hGFAPcreT2 mice was unclear.	*Mecp2*^tm2Bird^	hGFAPcreT2 mice were on a mixed FVB/N and C57BL/6 background and backcrossed on C57BL/6 for at least 8 generations. *Mecp2*^Stop/y^ mice were mixed 129P2 and C57BL/6 background and backcrossed on C57BL/6J.	Video tracking
Mantis et al., 2009	No	Yes	Yes	No	No	*Mecp2* null mice were originally from The Jackson Laboratory and then maintained in-house. The origin of wildtype mice was not clearly stated.	*Mecp2*^tm1Hzo^	*Mecp2* null mice were C576L/6J and 129S mixed background and backcrossed to C57BL/6J for at least 12 generations. Background not clearly stated for wildtype mice.	Automated system (open field test). Unclear method of data capture for light-dark assay.
McGill et al., 2006	No	Yes	Yes for ‘anxiety’ behavioural tests, but no for other tests/analyses	No	No	*Mecp2* null mice were presumably created in-house, since author Huda Zoghbi donated the mutation to The Jackson Laboratory. The origin of wildtype mice was not clearly stated	*Mecp2*^tm1Hzo^	*Mecp2* null mice were C576L/6J and 129SvEv mixed background and backcrossed to C57BL/6J for at least 12 generations. Background not clearly stated for wildtype mice.	Digiscan optical animal activity system (open field). Human observer (elevated plus maze and light-dark assay).
Mellios et al., 2014	No	Yes	Age range stated	No	No	Not stated	*Mecp2*^tm1Bird^	All mice were mixed 129P2 and C57BL/6 background and backcrossed on C57BL/6.	Data capture method was not stated.
Meng et al., 2016	No	Yes	Yes	No	Yes for behavioural experiments; not clearly stated for electrophysiology experiments.	Vglut2-Cre^+/−^ knock-in line was a gift from Dr. Brad Lowell; references were given regarding the other mouse lines, but their origin was not clearly stated.	*Mecp2*^tm1.1Bird^ and *Mecp2*^tm2Bird^	*Mecp2*^LSL/y^ and *Mecp2*^+/LSL^ mice were mixed C57BL6 and 129P2, backcrossed on 129S6SvEv. Vglut2-Cre^+/−^ mice were >99% FVB strain. *Mecp2*^flox+/y^ was mixed C57BL6 and 129P2 background. The remaining mice were created from crosses of the above. Background not clearly stated for wildtype mice.	Automated system (open field test and light-dark assay). Video tracking (elevated plus maze)
Mossner et al., 2020	No	Yes	Yes	No	Yes	All mouse lines were obtained from The Jackson Laboratory and crosses were performed in house.	*Mecp2*^tm1Bird/J^	*Mecp2*^flox+/y^;Dlx5/6-Cre mice were mixed CD1, C57BL/6 and 129P2 background. SOM-*Mecp2*^-/y^ mice were mixed 129S4Sv and C57BL/6 background. PV-*Mecp2*^-/y^ mice mixed C57BL/6 and 129P2 background Vip-*Mecp2*^-/y^ mice were mixed 129S4Sv and C57BL/6 background. Flox mice (used as ‘controls’) were mixed C57BL/6 and 129P2 background.	Video tracking
Nuber et al., 2005	No	Yes	No; authors chose to classify according to symptoms rather than age.	No	No	Not clear from methods, although the *Mecp2* null mice are presumed to have been created in house since the authorship includes Adrian Bird.	Presumed to be *Mecp2*^tm1Bird^	*Mecp2* null mice presumed to be mixed C57BL6 and 129P2 background. Background not clearly stated for wildtype mice.	N/A
Orefice et al., 2016	No	Yes for behavioural experiments; no for immunohistochemistry or electrophysiology	Yes	No	Yes	*Mecp2*^-/y^ and *Mecp2*^R306C^ mice were obtained from Michael Greenberg, Harvard Medical School (also obtainable through The Jackson Laboratory). *Mecp2*^f/y^, *Mecp2*^Stop^ and Emx1^Cre^ mice were obtained from The Jackson laboratory. Cdx2Cre mice were obtained from Eric Fearon. Advillin^Cre^ mice were obtained from Fan Wang, Duke University. Advillin^CreERT2^ mice were obtained from John Wood, University College London.	*Mecp2*^tm1.1Bird^, *Mecp2*^tm2Bird^ and *Mecp2*^R306^	Male and female mice were all of mixed C57BL/6J and 129/SvEv mixed backgrounds, except for *Mecp2* null and *Mecp2*^R306^ mice, which were on a C57BL/6J background.	Video tracking.
Ren et al., 2012	No	Yes	No	No	No	All mice were obtained from the Mouse Regional Resource Centre.	*Mecp2*^tm1.1Jae^	All mice were 129 and C57BL/6 mixed background.	N/A
Ricceri et al., 2011	No	Yes	Yes	No	Yes	*Mecp2* null mice were from The Jackson Laboratory. The origin of wildtype mice was not clearly stated.	*Mecp2*^tm1Hzo^	*Mecp2* null mice were of mixed C57BL/6J and 129SvEv background and backcrossed to C57BL/6J for at least 12 generations. Background not clearly stated for wildtype mice.	Video tracking.
Samaco et al., 2009	No	Yes	Age range stated	No	No	PET1-Cre mouse line was provided by Evan Deneris. The origin of other animals was not clearly stated, although author Huda Zoghbi is known to have created the *Mecp2*^tm1Hzo^ model, therefore *Mecp2* null mice have likely been produced in-house.	Not stated	*Mecp2*^-/y^ and their wildtype control – genetic background not clearly stated. TH-Cre animals were FVB/N background. PET1-Cre animals were C57BL/6 background. *Mecp2*^flox/+^ females of 129S6/SvEv background were bred with the Cre animals. TH-Cre; *Mecp2*^flox/y^ mice were therefore mixed FVB/N and 129S6/SvEv background and PET1-Cre; *Mecp2*^flox/y^ mice were mixed C57BL/6 and 129S6/SvEv background. The background of *Mecp2*^flox/y^ and wildtype controls was not clearly stated.	Digiscan optical animal activity system (open field test). Handheld computer (light-dark assay)
Su et al., 2015	No	Yes	Yes	No	No	Dlx5/6-Cre mice and *Mecp2*^Stop/+^ mice were from The Jackson Laboratory. *Mecp2*^flox/+^mice were from the Mutant Mouse Regional Resource Centre at UC Davis. All mice were backcrossed on C57BL/6 mice from the National Laboratory Animal Centre, Taiwan. The origin of wildtype mice was not clearly stated	*Mecp2*^tm1.1Jae^ and *Mecp2*^tm2Bird^	Dlx5/6-Cre mice were CD1 strain. *Mecp2*^flox/+^ mice were 129S4/SvJae, BALB/c and C57BL/6 mixed background. *Mecp2*^Stop/y^ mice were mixed C57BL/6 and 129P2 background. All mice were backcrossed to C57BL/6 for at least 10 generations. Background not clearly stated for wildtype mice.	Video capture.
Ure et al., 2016	No	Yes	No	No	Yes	Source not clearly stated.	*Mecp2*^tm2Bird^	*Mecp2*^lox−Stop/y^ mice were 129S6SvEvTac background, Viaat-Cre mice were FVB background and Viaat-Cre; *Mecp2*^lox−Stop/y^ mice were mixed 129S6SvEvTac and FVB background. Background not clearly stated for wildtype mice.	Automated system (open field test and light-dark assay). Video tracking (elevated plus maze).
Vigli et al., 2021	No	Yes	Yes	No	Yes	*Mecp2* mutant and wildtype mice were obtained from The Jackson Laboratory.	*Mecp2*^tm1Hzo^	All mice were mixed C57BL/6 and 129S background.	Video tracking
Wither et al., 2013	No	Yes	Age range stated	No	No	The Jackson laboratory.	*Mecp2*^tm1.1Bird^ and *Mecp2*^tm2Bird^	Mixed C57BL/6J and 129P2 background, backcrossed to C57BL/6.	Automated system (open field test). Human tracking (light-dark assay).
Zhang et at 2014	No	Yes	Yes	No	No	*Mecp2* null mice were obtained from The Jackson Laboratory and wildtype mice appear to have been obtained from SLAC laboratory Animal.	*Mecp2*^tm1Hzo^	*Mecp2* null mice were C57BL/6 and 129S mixed background, backcrossed to C57BL/6J for at least 12 generations. Wildtype controls appear to have been C57BL/6, although this is not explicitly stated.	Video tracking.
Zhang et al., 2016	No	Yes	The age-ranges were clearly stated, except for the age of animals where Western blot was performed.	No	Yes	Mice were obtained from The Jackson Laboratory and crosses performed in house. The origin of wildtype mice was not clearly stated.	*Mecp2*^tm1Bird^	*Mecp2*^flox/-^ mice were mixed C57BL/6J and 129P2 mixed background. Chat-IRES Cre mice were mixed C57BL/6J and 129S6 background. The paper notes that all mice were C57BL/6J strain and therefore the above mice were presumably backcrossed to C57BL/6J. The strain of wildtype mice was not explicitly stated.	The behaviour in the elevated zero maze was videotaped and the data capture method for the light-dark assay, open field test and elevated plus maze was not stated.
Zhou et al., 2017	No	Yes	Yes	No	Yes	All mice were obtained from The Jackson Laboratory and crosses performed in house. The wildtype mice were created as part of the genetic cross.	*Mecp2*^tm2Bird^	*Mecp2*^Stop/y^ mice were mixed C57BL/6J and 129P2 mixed background. Chat-IRES Cre mice were mixed C57BL/6J and 129S6 background. Both strains were backcrossed to C57BL/6J.	Automated system (open field test). Video tracking (elevated plus maze).

### 3.2 Circuitry regions and cellular subtypes where loss of MeCP2 altered anxiety behaviour

#### 3.2.1 Macrocircuitry regions

Loss of MeCP2 in the peripheral sensory ganglia has been associated with increased anxiety behaviour. Mice with this mutation had enhanced sensitivity to tactile stimuli when compared to controls. It is important to note that mice would have a greater anxiety response to touch than humans. However, the authors comment that other studies have shown that humans who have had sensory deprivation in early life have increased anxiety (Orefice et al., 2016). Deletion of *Mecp2* in the amygdala was associated with an increase in anxiety behaviour in male mice (Adachi et al., 2009; Gemelli et al., 2006). There is evidence for deletion of *Mecp2* in the nucleus accumbens and prefrontal cortex being associated with increased anxiety (*Mecp2* was deleted from multiple anatomic regions, therefore the relevance of individual regions is not clear) (Gemelli et al., 2006).

#### 3.2.2 Cellular subtypes

Selective reintroduction of *Mecp2* in astrocytes of *Mecp2* null mice was associated with anxiety behaviour being more typical of behaviours of control mice (although interpretation is complex as the null mice showed mixed anxiety behavioural changes compared to control) (Lioy et al., 2011).

### 3.3 Circuitry regions and cellular subtypes where loss of MeCP2 had mixed effects on anxiety behaviour

#### 3.3.1 Macrocircuitry regions

Lower MeCP2 expression in the hippocampus in *Mecp2*^+/−^ mice was associated with lesser anxiety behaviour. However, expression of MeCP2 was not measured in anxiety-associated brain regions other than the hippocampus, making interpretation of these results difficult (Wither et al., 2013). In contrast when *Mecp2* was removed from the forebrain (inclusive of hippocampus), there was an increase in anxiety behaviour (Gemelli et al., 2006). Sim-1 is a gene regulatory element required for the development of neurons in the paraventricular, supraoptic, and posterior hypothalamic nuclei, and in the nucleus of the lateral olfactory tract of the amygdala. Deletion of *Mecp2* in Sim1-expressing neurons led to a decrease in MeCP2 expression in these regions. This did not result in a reliable difference in ‘anxiety’ behaviour compared to controls (Fyffe et al., 2008).

#### 3.3.2 Cellular subtypes

Altered MeCP2 function in cholinergic (Zhou et al., 2017; Zhang et al., 2016), glutamatergic (Meng et al., 2016; Goffin et al., 2014) and GABAergic (Chao et al., 2010; Ure et al., 2016) neurons resulted in a variable pattern of changes in anxiety behaviour. Mice with specific removal of *Mecp2* from tyrosine hydroxylase-expressing dopaminergic (DA)/noradrenergic (NA) neurons showed no difference in anxiety behaviour compared to controls (Samaco et al., 2009). Rescue of *Mecp2* in catecholaminergic neurons normalised anxiety behaviour in male and female *Mecp2*-deficient mice (Lang et al., 2012). However, there was no significant effect on anxiety behaviour when *Mecp2* was rescued in the dorsal striatum of *Mecp2* deficient male and female mice (Su et al., 2015). Overall, this implies that if MeCP2 function in DA/NA neurons does affect anxiety behaviour, that this occurs via a specific subtype of catecholaminergic neuron and/or particular brain region.

In adult mice, microglial cells develop within the bone marrow and migrate to the brain (in addition to slow proliferation of microglia within the CNS). *Mecp2*^+/−^ mice were irradiated to kill resident microglia and then given a bone marrow transplant from wildtype mice. Behavioural testing indicated that transplantation (intended to give rise to normal microglia) resulted in anxiety behaviour reducing to wildtype levels (Derecki et al., 2012). A later study used *Mecp2* null mice and replicated these methods. Anxiety behaviour was not explored, but other symptoms were not reversed through introduction of wildtype microglia, suggesting that anxiety behaviour may also not have been altered (Wang et al., 2015).

### 3.4 *Circuitry regions and cellular subtypes where loss of MeCP2 did not alter anxiety behaviour*

#### 3.4.1 Cellular subtypes

Specific loss of MeCP2 in GABAergic interneurons – parvalbumin, somatostatin, vasoactive intestinal peptide (separate or combined) - was not associated with a change in anxiety behaviour (Ito-Ishida et al., 2015; He et al., 2014; Mossner et al., 2020).

Mice in which *Mecp2* was deleted in PET1-expressing serotonergic neurons showed no difference in anxiety behaviour compared to controls. (Samaco et al., 2009). *Mecp2* null mice were found to have lower 5-HT7R (serotonin receptor) density in the cortex and hippocampus compared to wildtype. Intraperitoneal injection of LP-211 (a 5-HT7R selective agonist) in *Mecp2* null mice was associated with increased cortical 5-HT7R density, but had no clear effect on anxiety behaviour (De Filippis et al., 2014). Serotonin is a key neurotransmitter in anxiety disorders, so this apparent lack of effect on anxiety behaviour is of note. It has been shown that MeCP2 is involved in enhancing transcription of 5-HT1AR (Philippe et al., 2018) and so it is possible that either MeCP2 does not modulate 5-HT7 production or that a greater concentration of 5-HT has less impact if there is lower receptor expression. In addition, studies have shown that administration of fluoxetine (a selective serotonin reuptake inhibitor, SSRI) resulted in raised MeCP2 levels in GABAergic neurons (Cassel et al., 2006) and increased MeCP2 expression in the prefrontal cortex but not the CA3 region of the hippocampus (Villani et al., 2021), highlighting that MeCP2 function within serotonergic neurons may be different to the effects of serotonin itself.

Cannabidiolic acid administration did not reduce anxiety in *Mecp2* null mice (Vigli et al., 2021). This contrasts with an earlier study that reported cannabidivardin improved symptoms (anxiety behaviour not assessed) in *Mecp2* null mice (Vigli et al., 2018), so it is possible that cannabinoid receptors may be relevant to anxiety in Rett syndrome.

### 3.5 Treatment studies that may provide further insights into these mixed effects on anxiety behaviour

Electrophysiological analysis of isolated cortical cell membranes of *Mecp2* null mice showed reduced GABA and glutamate currents relative to those from *Mecp2* null mice that had been administered mirtazapine prior to analysis. However, administration of mirtazapine and desipramine to *Mecp2* null mice did not result in a clear change in anxiety behaviour (Bittolo et al., 2016). *Mecp2*^+/−^ mice initially showed a reduction in anxiety compared to wildtype and anxiety apparently increased with mirtazapine. However, this was subsequently attributed to increased whisker sensitivity altering the behaviour of the mice in the closed arms of the assay. Albeit whisker (sensory) sensitivity may be relevant to anxiety, in line with earlier findings (Orefice et al., 2016)). There was a reduction in expression of parvalbumin positive neurons (fast-firing GABAergic interneurons) in the barrel cortex and basolateral amygdala in *Mecp2*^+/−^ mice; expression was increased in the barrel cortex but not the amygdala following mirtazapine treatment, suggesting a possible mechanism for the increased sensitivity (Flores Gutiérrez et al., 2020).

The mechanism of action of deep-brain magnetic stimulation (DMS) is largely unknown, though it may modulate the balance of excitatory and inhibitory circuits. Treatment with DMS for 5 months showed some reduction in anxiety behaviour of *Mecp2* null mice to wildtype levels (Zhang et al., 2014). Deep brain stimulation (DBS) also appeared to reduce anxiety behaviour in *Mecp2*^+/−^ mice. However, the same effect was found with sham DBS, indicating handling and exposure as the reason for decreased anxiety (Hao et al., 2015).

### 3.6 Molecular changes that potentially associate with the anxiety behavioural changes/physiologic response

#### 3.6.1 MeCP2 effects on circuitry connections and molecular changes that are cell type and region specific

Loss of MeCP2 in the peripheral sensory ganglia may cause loss of the β3 unit of the GABA_A_ receptor in these regions, resulting in increased peripheral nerve conduction, which may act as a mechanism for the increased anxiety behaviour (Orefice et al., 2016). Excitatory projections from the cortex and thalamus to the lateral nucleus of the amygdala (LA) are involved in fear acquisition. MeCP2 specifically affected the functioning of the cortico-LA pathway (not the thalamo-LA pathway) through effects on stabilisation of AMPA receptors and synaptic elimination and maturation, highlighting a potential mechanism of anxiety in Rett syndrome, since there is an overlap between fear and anxiety circuitry (Gambino et al., 2010). (Gambino et al., 2010).

Loss of MeCP2 in cholinergic neurons in the basal forebrain led to decreased expression of choline acetyltransferase (ChAT). This was associated with reduced excitability of the cholinergic neurons, leading to downstream increased excitability of hippocampal pyramidal neurons. According to anxiety behavioural changes, it appears that these effects of MeCP2 were pathway-specific (Zhang et al., 2016). By turn, choline supplementation significantly increased ChAT activity in *Mecp2* null and wildtype mice, and reduced *Mecp2* null anxiety levels to those of wildtype mice (Ricceri et al., 2011). Choline supplementation in mice with specific loss of MeCP2 in excitatory neurons of the forebrain was associated with decreased anxiety and increased neurite length, number of dendritic branches and dendritic spine density, in the basal forebrain suggesting further molecular changes resulting from MeCP2 function in cholinergic neurons (Chin et al., 2019).

Tyrosine hydroxylase (Th) and tryptophan hydroxylase 2 (Tph2) are rate-limiting enzymes in the production of DA/NA and 5-HT, respectively. MeCP2 may be necessary for their production as MeCP2 was found to bind to the Th and Tph2 promoters and there were reduced RNA levels for Th and Tph2 in *Mecp2* null mice relative to wildtypes. Again this appears to be region-specific, since the expression of Th was lower in the locus coeruleus, midbrain containing substantia nigra and ventral tegmental area, but not the medulla, in *Mecp2* null mice relative to wildtypes and Tph2 expression was lower in the hindbrain containing raphe nucleus B1-3, but not in the hindbrain region containing raphe nucleus B4-9, in *Mecp2* null mice relative to wildtypes. These changes in enzyme expression were associated with reduced levels of monoamines (Samaco et al., 2009).

#### 3.6.2 Influence on cell signalling pathways

IGF-1 and BDNF both act via the phosphoinositide 3-kinase (PI3K)/Protein kinase B (Akt) pathway and the extracellular signal-regulated kinase (ERK) pathways. Lower serum IGF-1 (Mellios et al., 2014) and lower hippocampal BDNF expression (Kondo et al., 2016) were found in *Mecp2*^+/−^ mice compared to wildtype mice. Increasing the serum IGF-1 levels (via clenbuterol treatment, a beta adrenergic agonist (Mellios et al., 2014)) and environmental enrichment (Kondo et al., 2016) resulted in anxiety behaviour returning to wildtype levels. Intraperitoneal rhIGF-1 treatment resulted in an increase of anxiety behaviour of *MeCP2* null mice to wildtype levels (Castro et al., 2014). Overall, these results indicate that modulation of IGF-1 and BDNF expression by MeCP2 may contribute to anxiety behaviour.

#### 3.6.3 Possible changes to metabolic activity of CNS cells

Alterations in access to food in *Mecp2* null mice suggested an improvement in anxiety. Mice fed a ketogenic diet or standard diet with caloric restriction had reduced anxiety compared to those given standard diet. However, the mice on restricted diets were more active as they persistently searched for food, wildtype mice on a similar restricted diet also showed a decrease in anxiety behaviour. This shows little evidence for the effects of cellular metabolic activity on anxiety behaviour or evidence that MeCP2 might affect metabolic activity (Mantis et al., 2009).

#### 3.6.4 Altered HPA axis function

mRNA for FKBP5 (a negative feedback regulator of the glucocorticoid receptor) and serum and glucocorticoid-inducible kinase (Sgk) levels were upregulated in whole-brain samples from *Mecp2* null mice compared to wildtype, however there was no difference in levels in the basolateral amygdala, suggesting MeCP2's modulation of their expression is region-specific (Adachi et al., 2009; Nuber et al., 2005). *Mecp2* null mice in environmentally enriched housing showed increased mRNA expression of Sgk1in the hypothalamus and cortex that was associated with anxiety returning to wildtype levels, identifying a possible role for Sgk1 regulation in anxiety. (Kerr et al., 2010).

MeCP2 regulation of corticotropin releasing hormone (CRH) expression differs between brain regions and between male and female mice: there was elevated CRH in the paraventricular nucleus of the hypothalamus, central amygdala and the bed nucleus of the stria terminalis (McGill et al., 2006) of *Mecp2* null compared to wildtype mice, whilst *Mecp2*^+/−^ mice had lower expression of CRH mRNA in the hypothalamus than wildtype mice (Kondo et al., 2016). CRH mRNA expression in the basolateral amygdala was not significantly different in mice without MeCP2 in the amygdala, compared to controls (Adachi et al., 2009). The HPA axis was normal in *Mecp2* null mice and so increased CRH expression (in certain regions) was identified as a key source of HPA axis dysfunction in *Mecp2* null mice (McGill et al., 2006). However, CRH1 antagonist administration significantly reduced anxiety behaviour of wildtype, but not *Mecp2* null mice, whilst under restraint stress. Wildtype and *Mecp2* null mice showed an irregular breathing pattern when restrained using a small restraint chamber, indicating a possible anxiety response. CRHR1 antagonist administration resulted in a closer to normal breathing pattern in the wildtype mice in the small chamber but notably affected breathing in *Mecp2* null mice in both chambers (Ren et al., 2012).

Physiological responses to stress, assessed through serum corticosterone levels were equivalent in *Mecp2* null and wildtype mice at baseline and following 5–15 ​min of restraint stress (McGill et al., 2006; Nuber et al., 2005). However, this response altered after 30–60 ​min of restraint, at which point higher serum corticosterone was measured in *Mecp2* null mice (McGill et al., 2006; Ren et al., 2012). *Mecp2* null mice showed a greater increase in anxiety behaviour when moved from a large to a small chamber (restraint stress) compared to wildtype mice (Ren et al., 2012). Environmental enrichment is associated with a reduction in corticosterone levels in *Mecp2*^+/−^ mice and anxiety behaviour being similar to wildtype (Kondo et al., 2016).

### 3.7 Environmental changes overlap with/modify the effects of genetic loss of MeCP2

Environmental enrichment normalised some anxiety behaviours in *Mecp2* null mice. Behavioural testing was performed in two mouse strains: 129/SvJ and mixed 129/SvJ and C57BL/6J background. 129/SvJ mice are known to perform poorly in the elevated plus maze (Kerr et al., 2010). Environmental enrichment normalised anxiety in *Mecp2*^+/−^ mice (Kondo et al., 2016). Environmentally enriched *Mecp2* null mice had significantly lower mRNA expression for synaptophysin and post-synaptic density-95 protein in the hypothalamus, and lower mRNA expression for syntaxin 1A and synaptotagmin in the cortex, when compared to standard housed *Mecp2* null mice (Kerr et al., 2010).

Maternally-derived cortisol was shown not to affect anxiety behaviour of *Mecp2* null and wildtype pups, or their response to restraint stress. Despite this, changes were found in mineralocorticoid receptor (MR) mRNA expression, which was increased in both genotypes in mice with mothers who drank cortisol solution; this effect was more marked in the *Mecp2* null mice. Glucocorticoid receptor (GR) mRNA expression was increased in *Mecp2* null mice with mothers who drank cortisol solution compared to *Mecp2* null mice without cortisol supplementation. (De Filippis et al., 2013).

Behavioural testing of *Mecp2*^+/−^ and wildtype mice separated from their mothers (intended to induce early life stress) indicated that *Mecp2*^+/−^ mice were less anxious and that maternal separation did not alter this. However, when exposed to a more anxiogenic paradigm – elevated plus maze under bright-light conditions – maternal separation resulted in both wildtype and *Mecp2*^+/−^ mice being less anxious than their non-separated counterparts. Although maternal separation is usually used to induce early life stress, the authors noted that *Mecp2*^+/−^ mothers (mothers of both wildtype and *Mecp2*^+/−^ offspring in this study) tend to be neglectful and cannibalistic and C57BL/6 mice (the background in this study) also tend to be neglectful. Therefore, separation may have enabled the mice to become more resilient than the mice who stayed with the mothers. (Abellán-Álvaro et al., 2021).

## 4 Discussion

### 4.1 Summary of findings

Many of the included studies illustrated effects on anxiety behaviour through use of mouse models with specific loss/reintroduction of *Mecp2* from specific brain regions or cell subtypes. Loss/reintroduction of *Mecp2* to some anxiety macrocircuit regions was associated with a change in anxiety behaviour, but for the majority of studies with loss/reintroduction of *Mecp2* from particular cell subtypes, there were mixed effects on anxiety behaviour. Fig. 2, panel A summarises the brain regions identified as contributing/not contributing to anxiety in models of Rett syndrome and maps them onto the regions currently recognised to have a role in anxiety processing (Calhoon and Tye, 2015). There is evidence (Orefice et al., 2016) that the anxiety response could be modulated as early as the sensory detection stage. Fig. 2, panel B summarises the cell types identified as contributing/not contributing to anxiety in models of Rett syndrome. The mixed results found within some studies (Zhou et al., 2017; Zhang et al., 2016; Chao et al., 2010; Ure et al., 2016; Samaco et al., 2009; Lang et al., 2012; Su et al., 2015) may reflect: variability in the validity of the anxiety behavioural tests (discussed in detail in section 4.2); the specificity of MeCP2 function within particular cell types; the fact that removal of *Mecp2* from a cell subtype throughout the brain may mask the effects of that cell in a specific microcircuit; or may arise from the complexity of anxiety microcircuits (making a change to one neuronal or neurotransmitter subtype may not cause a definitive change to the overall circuit). If this last aspect were the case, there would be implications for anxiolytic drug treatments, which at present tend to target single or only few neurotransmitters. Inflammation is increasingly regarded as a contributor to mental disorders. Although the relevance of MeCP2 function in microglia is unclear (Derecki et al., 2012; Wang et al., 2015), there is evidence for an altered systemic inflammatory profile and for altered microglial activation (which can lead to increased release of proinflammatory molecules) in Rett syndrome (Jin et al., 2017) suggesting possible routes for neuroinflammation contributing to anxiety symptoms.

Loss of MeCP2 in certain brain regions has been associated with reduced neurotransmitter (Orefice et al., 2016; Zhang et al., 2016; Samaco et al., 2009; Ricceri et al., 2011) and growth factor expression (Kondo et al., 2016; Mellios et al., 2014; Castro et al., 2014), loss of neurotransmitter receptor subunits (Orefice et al., 2016), reduced stabilisation of AMPA receptors (Gambino et al., 2010), and alterations to synapses (Gambino et al., 2010).

There is evidence for MeCP2 loss having an impact on regulation of the HPA axis, and mechanisms are beginning to be understood (McGill et al., 2006; Adachi et al., 2009; Nuber et al., 2005; Kerr et al., 2010; Ren et al., 2012). There is evidence for increased FKBP5 mRNA expression in whole brains of null mice and CRH expression may be altered, although this appears to vary between brain regions and between male and female mice (McGill et al., 2006; Kondo et al., 2016; Nuber et al., 2005). *Mecp2* null mice show elevated serum corticosterone levels at a delayed timepoint following restraint stress (McGill et al., 2006; Ren et al., 2012).

### 4.2 Validity of the pre-clinical studies

*Mecp2* mouse models have reasonable construct validity, as the majority of humans with Rett syndrome have *MECP2* mutations. However, most mouse models are null for *Mecp2*; this is representative of large deletions in humans, which account for only 10% of cases. Although mouse models with conditional deletion of the *Mecp2* gene are useful in understanding function of MeCP2 in individual cell subtypes, they do not recapitulate Rett syndrome, since they do not represent the global loss of MeCP2 and therefore have limited translational value. In models where there is conditional deletion of the *Mecp2* gene in the targeted region, it is notable that they do not always result in complete loss of MeCP2 expression in that region (Adachi et al., 2009; Gemelli et al., 2006).

Only 11/38 of the studies included female mice (all found within Table 1), whereas Rett syndrome almost exclusively affects girls (Katz et al., 2012). This is of particular significance in the study of anxiety disorders, since the prevalence of anxiety disorders in the general population is twice as high in women compared to men (Bandelow and Michaelis, 2015) and sex-dependent effects on anxiety and stress-vulnerability have been found in *Mecp2*-deficient mice (Philippe et al., 2018; Cosentino et al., 2021).

As shown in Table 2, *Mecp2* mouse models have differing genetic backgrounds and have been obtained from different sources. This makes comparison between studies and reproducibility more difficult. Although it has been shown that certain mouse models may show increased anxiety behaviour compared to others, there is no clear pattern amongst the studies reviewed in this paper (Samaco et al., 2013). The effect of environmental enrichment on anxiety in mice with *Mecp2* mutations emphasises that standardised animal husbandry practices are important in securing the reproducibility of results (Katz et al., 2012; Kondo et al., 2016; Kerr et al., 2010) and the maternal separation study indicates that maternal temperament may affect anxiety (Abellán-Álvaro et al., 2021).

There is debate as to the validity of mouse anxiety behavioural experiments. The behavioural experiments discussed in this paper best approximate to generalised anxiety disorder and specific phobias. These tests are imperfect, but there is no ‘gold standard’ anxiety behavioural experiment. Tests similar to the elevated plus maze and open field test have been performed in humans and behaviour within these tests is comparable between mouse and human. However, further work is needed to understand if the neurobiological anxiety mechanisms are comparable between mouse and human in these paradigms (Bach, 2022). One key limitation of mouse behavioural experiments in the context of Rett syndrome mouse models is the motor impairment component of the phenotype. As anxiety behavioural experiments all rely on locomotion, this presents a clear a problem in interpretation. Measures such as ultrasonic vocalisation may be a useful alternative that does not rely on motor function (Mun et al., 2015).

Although decreased time in the open arm and increased time in the closed arm of the elevated plus maze are typically interpreted as signs of increased anxiety in mice, it has been shown that female *Mecp2*^tm1.1Bird^ mice avoid the closed arms due to whisker hypersensitivity, rather than true preference for the open arm (Flores Gutiérrez et al., 2020). This potentially affects the interpretation of three of the papers involving female mice (Kondo et al., 2016; Meng et al., 2016; Abellán-Álvaro et al., 2021). It would be important to clarify further whether this response is found across different *Mecp2* mutations, mouse genders and with loss of MeCP2 from specific cells/anatomical regions. It also suggests that the open field test and light-dark assay may be preferable tests for quantifying anxiety behaviour in Rett syndrome mouse models, compared to the elevated plus maze or elevated zero maze (Lioy et al., 2011; Ure et al., 2016; Flores Gutiérrez et al., 2020).

A diverse number of behavioural tests and variety of behavioural measures have been used in the studies within the review. For example, some studies measured latency to enter the open area of the elevated plus maze and others measured the time spent in the open area. This is a further factor that makes comparison between studies difficult. Physiological measures, such as heart rate may be considered as a more objective and translatable measure of anxiety response and it has been shown that physiological changes occur when mice are in the elevated plus maze (Okonogi et al., 2018).

### 4.3 Anxiety mechanisms identified within human studies

#### 4.3.1 Neurotransmitters and their metabolites

Prior to the identification of *MECP2* mutation as the main cause of Rett syndrome, early research into the pathology of the syndrome identified that reduction of levels of biogenic amines and their metabolites was a common feature; however biogenic amines were not correlated with anxiety levels (He et al., 2014; Roux and Villard, 2010). Individuals with the pArg168X mutation (associated with more severe overall symptoms of Rett syndrome) had significantly lower cerebrospinal fluid (CSF) 5-hydroxy indole acetic acid (5-HIAA) levels than individuals with the pArg133Cys (associated with less severe global impairment) mutation or controls (Samaco et al., 2009). RSBQ and ADAMS anxiety scores have significant (or trend towards) inverse correlation with level of global impairment (Barnes et al., 2015; Anderson et al., 2014; Robertson et al., 2006). These findings suggest that lower CSF 5-HIAA levels are associated with lower anxiety levels in Rett syndrome (Samaco et al., 2009; Barnes et al., 2015). A study has not yet been done exploring the efficacy of SSRIs in individuals with Rett syndrome, however as part of the Rett Syndrome Natural History Study, parents have reported that they observed improvement in anxiety symptoms with SSRI treatment (Buchanan et al., 2022).

#### 4.3.2 HPA axis

Despite there being a number of pre-clinical studies suggesting a role for MeCP2 affecting HPA axis function, there have been few clinical studies exploring this. Total free urinary cortisol/24 ​h correlates well with free plasma cortisol levels (El-Farhan et al., 2017). A study involving 10 females with Rett syndrome (stage III-IV) compared to age- and sex-matched controls showed no significant difference in total urinary cortisol/24 ​h (Motil et al., 2006). In healthy individuals there is a 90% reduction in evening cortisol compared to morning concentration. An exploratory study of 30 individuals with Rett syndrome identified that in individuals with mutations leading to less severe impairment, the diurnal variation was more pronounced (closer to normal) than individuals with mutations leading to more severe global impairment (Byiers et al., 2020). Further study, including correlation of cortisol with RSBQ, will be needed to understand the impact of MeCP2 on the HPA axis.

#### 4.3.3 Growth factors

Although preclinical studies suggest a role for IGF-1 in anxiety behaviour, clinical trials for IGF-1 have produced a complex picture, discussed by Singh and Santosh (2018). IGF-1 treatment is due to be studied further in a Phase 3 trial (Neul et al., 2022). A post-mortem histology study suggested that BDNF expression is region-specific and that levels were higher in the cerebellum in Rett syndrome patients (Pejhan et al., 2020a) although a study by the same group using ELISA (quantifying protein levels) suggested BDNF levels were lower in all regions (Pejhan et al., 2020b); more detailed study of BDNF and IGF-1 expression in anxiety regions may be helpful for understanding its mechanism in Rett syndrome.

#### 4.3.4 Gut-brain axis

Gastrointestinal symptoms are a common feature of Rett syndrome. Differences in the gut microbiome have been reported between Rett syndrome patients, but these did not correlate with differences in anxiety symptoms (as measured by the Gastrointestinal Health Questionnaire, validated tool for use in Rett syndrome patients), however it is possible that confounding factors affect interpretation of this correlation (Thapa et al., 2021).

#### 4.3.5 Sex-hormone influence

Fifteen out of twenty-one young women with Rett syndrome reported that premenstrual syndrome was a problem, and eight women described anxiety as being one of the premenstrual symptoms, suggesting their mood was modulated by hormonal changes (Hamilton et al., 2012).

#### 4.3.6 Potential environmental influence

Hospital Anxiety and Depression Scales (HADS) (Zigmond and Snaith, 1983) were completed by mothers of individuals with Rett syndrome who were found to have greater anxiety, but not depression, than a British normative sample. Siblings of those with Rett syndrome had similar emotion regulation (according to the Strengths and Difficulties Questionnaire) (Goodman, 1997) to the normal population. An increased severity of the RSBQ score (in the child with Rett syndrome) was correlated with a higher level of maternal stress and anxiety (Cianfaglione et al., 2015). Unlike in preclinical studies, environmental enrichment did not alter anxiety symptoms (Downs et al., 2018).

#### 4.3.7 Summary

When combined with findings from a previous review of autonomic dysregulation in Rett syndrome (Singh and Santosh, 2018), the findings described within the current review indicate that there are multiple potential mechanisms contributing to anxiety in Rett syndrome. As identified from the preclinical studies, further study is needed to understand the precise mechanisms of serotonin in contributing to anxiety in Rett syndrome. It appears that certain *MECP*2 mutations are associated with greater anxiety symptoms.

### 4.4 Epigenetics

#### 4.4.1 Within rett syndrome

Although Rett syndrome is a genetic disorder, the mutation of *MECP*2 has epigenetic consequences, which are complex (Marano et al., 2021). This MeCP2 dysfunction can interact with other epigenetic mechanisms; for example there may be differing levels of DNA methylation between individuals with Rett syndrome, adding another layer to the way MeCP2 can function (Marano et al., 2021). Pre-clinical and clinical studies within this paper highlight potential environmental influences (with likely epigenetic effect) which may contribute to anxiety in Rett syndrome (De Filippis et al., 2013; Abellán-Álvaro et al., 2021; Cianfaglione et al., 2015).

#### 4.4.2 Rett syndrome may inform understanding of the epigenetics of anxiety

**Fig. 3:**
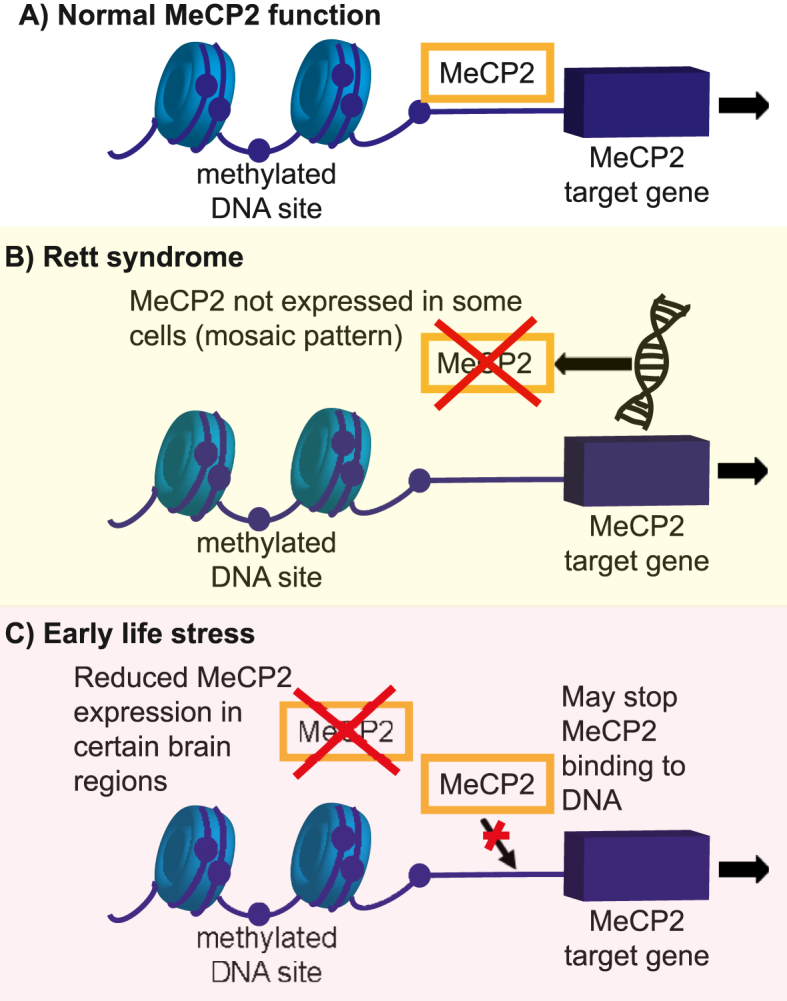
Proposed mechanistic similarity between Rett syndrome and early life stress. A) illustrates the normal function of MECP2 in binding to DNA and influencing gene transcription. B) highlights that MECP2 cannot have this function in Rett syndrome since it is not produced due to mutations of the MECP2 gene. C) illustrates results from 2 pre-clinical studies that identified that early life stress resulted in reduced MECP2 expression in certain brain regions (Lewis et al., 2016) and reduced binding of MECP2 to DNA (Murgatroyd et al., 2009). The figure illustrates the similarly limited MECP2 function in B and C.

Epigenetic modulators, including MeCP2, are increasingly recognised as the mechanism by which development (and later life changes) can be affected by environment. Early life stress is a key environmental influence that predisposes individuals to mental health disorders, including anxiety disorders (Gottschalk and Domschke, 2016; Bearer and Mulligan, 2018; Schiele et al., 2020). Preclinical studies have demonstrated different mechanisms linking reduced MeCP2 function with early life stress (Murgatroyd et al., 2009; Lewis et al., 2016) and these are depicted in Fig. 3. This mechanism is of interest since it suggests mechanistic overlap with Rett syndrome and that downstream cellular and molecular anxiety mechanisms of Rett syndrome could also be relevant to those with anxiety and a history of early life stress. Further, *MECP2* mutation and epigenetic modification leading to altered *MECP2* gene expression is reported in patients with autism spectrum condition (ASC) and schizophrenia (Qiu, 2018; Ciernia and LaSalle, 2016; Richetto and Meyer, 2021). In a preclinical model, specific methylation of the *Mecp2* promotor in the hippocampus was sufficient to reduce *Mecp2* expression and induce most ASC-like behaviours (Lu et al., 2020). ASC and schizophrenia have been associated with aberrant neuronal circuitry and anxiety (Qiu, 2018).

Epigenetic mechanisms of anxiety have previously been reviewed (Gottschalk and Domschke, 2016) and the importance of developing biomarkers for the epigenetic effects of stress to enable individualised treatment has been emphasised (Gottschalk et al., 2020). Peripheral biomarkers (often blood and saliva) can only provide limited understanding of epigenetic changes within the CNS. Rett syndrome models may provide a method to develop epigenetic biomarkers – such as blood, saliva, and possibly cerebrospinal fluid – that may correlate with CNS mechanisms and anxiety symptoms. This could be used to enhance understanding of anxiety in those exposed to early life stress or with ASC, in addition to guiding anxiety treatment in Rett syndrome (Bearer and Mulligan, 2018).

### 4.5 Translation to therapeutic applications

*Mecp2/MECP2* mutations result in comparable effects in mice and humans. This increases the likelihood that biological mechanisms are conserved between species and that these may have value as translational models for developing targeted drug treatments.

At present drug treatments for anxiety disorders are largely limited to SSRIs and serotonin and noradrenaline reuptake inhibitors (SNRIs). Understanding the molecular and cell- and region-specific effects of MeCP2 loss will be important in developing effective treatments for anxiety in Rett syndrome. The mechanisms identified in this review highlight the potential breadth of targets, for example the sensory system, a wider range of neurotransmitters and regulation of the HPA axis. The current range of investigations do not allow us to identify reliable potential treatment targets, but potentially compounds targeting astrocytic functions may be relevant (Lioy et al., 2011). Results from pre-clinical and clinical studies seem to suggest that only specific serotonergic agents may be effective (Samaco et al., 2009; De Filippis et al., 2014; Barnes et al., 2015).

Given the potential overlapping mechanism of Rett syndrome and early life stress, treatments for anxiety in Rett syndrome may also be effective in those with anxiety and a history of early life stress.

Anxiety in patients with ASC is often difficult to treat but there have been few studies of the efficacy of pharmacotherapy. The current evidence from studies of children and adolescents is insufficient to support treatment with SSRIs (Stepanova et al., 2017). This suggests the need for an alternative innovative approach to treating anxiety symptoms and disorders, possibly linked to MeCP2 and its isoforms (Good et al., 2021).

As well as anxiety, disordered breathing is a common feature in Rett syndrome. Although anxiety can worsen respiratory function, it is possible that altered respiratory function can exacerbate anxiety (Ren et al., 2012). Within the field of anxiety research, the 7.5% CO_2_ inhalation experimental medicine model is well established. In this model humans inhale 7.5% CO_2_ over 20 ​min, which induces subjective and autonomic responses and neurocognitive changes that are comparable to the features of generalised anxiety disorder. The mechanisms are only partially understood (Baldwin et al., 2017). Given that carbon dioxide/acid sensing is involved in breathing and the CO_2_ anxiety model, this prompts consideration of an overlap between anxiety and breathing mechanisms in Rett syndrome, for example via similar cellular or molecular sensing mechanisms. Exploration of these factors may extend knowledge of contributing mechanisms in the CO_2_ model and so inform understanding of overall mechanisms of anxiety.

### 4.6 Conclusion and next steps

Rett syndrome is a complex disorder with multiple intertwining cellular and systems abnormalities. The combined findings of pre-clinical and clinical studies show that progress is being made towards improved understanding of anxiety mechanisms in Rett syndrome.

Future steps for pre-clinical work will need to include greater use of female mice within studies, clarification of whether whisker sensitivity (affecting interpretation of behaviour in the elevated plus maze) is found across Rett syndrome models, inclusion of tests that are not affected by motor impairment (such as ultrasonic vocalisation) and inclusion of physiological markers (corticosterone and heart rate) that are quantifiable and translatable. Standardisation of anxiety tests would enable improved comparison between studies. Given that environmental factors may modify anxiety, standard environment should be used as well as consideration of maternal temperament. In terms of added mechanistic understanding, it will be helpful to develop understanding about the effects of *Mecp2* mutation on specific anxiety-circuitry connections.

Given the importance of anxiety as a symptom within Rett syndrome, clinical studies should increasingly aim to include anxiety measures, including RSBQ. The HPA axis in individuals with Rett syndrome is an area that also requires increased study.

Improved understanding of anxiety mechanisms in Rett syndrome (which may include development of biomarkers to identify the mechanisms present within an individual, given the heterogenous nature of the syndrome) could enable more effective treatments to be developed. Importantly, the known *MECP2* mutation means that there is the potential for translation from mouse models to human therapeutic applications. Improved understanding of potential overlapping mechanisms between Rett syndrome, autism and early life stress may allow more effective anxiolytic treatments to be developed across these disorders.

## Funding

Bethan Impey worked on this review whilst in position as an NIHR funded Academic Clinical Fellow.

## Declaration of competing interest

The authors declare the following financial interests/personal relationships which may be considered as potential competing interests: BI has a small number of stocks and shares in GlaxoSmithKline. TAN has no conflicts of interest to declare. DSB is Medical Patron of Anxiety UK, and President-Elect of the British Association for Psychopharmacology. He receives an editor fee from Wiley, for his editorship of *Human Psychopharmacology*. He was an unpaid consultant for Idorsia in July 2020.
